# Green production of titanium dioxide nanometric particles through electrolytic anodic dissolution of titanium metal

**DOI:** 10.1007/s11356-022-23766-3

**Published:** 2022-11-05

**Authors:** Hassan H. Shaarawy, Hala S. Hussein, Nabila H. Hussien, Ghada A. Al Bazedi, Salwa I. Hawash

**Affiliations:** 1 Chemical Engineering & Pilot Plant Dep, Engineering Research and Renewable Energy Institute, National Research Centre (NRC), Cairo, 12311 Egypt; 2 Center of Applied Research On the Environment and Sustainability(CARES), School of Science and Engineering, The American University, Cairo, Egypt

**Keywords:** Nanometric particles, Titanium dioxide, Anodic dissolution

## Abstract

Nanometric titanium derivatives such as hydroxide and dioxide compounds have a great attention because they are significant industrial material of commercial importance and applications in photocatalyst, semiconductors, and wastewater treatment. The present investigation gives the results of anodic dissolution preparation of titanium hydroxide nanometric particles followed by calcination for complete conversion to nanometric titanium dioxide product. The optimum conditions for the anodic dissolution of titanium metal were pH 4, C.D. 65 mA/cm^2^, 25 °C, 150 rpm, electrode gap distance 3 cm, and NaCl 3 g/l for electrolysis time 240 min and thermally calcinated at 600 °C for 240 min., to reach complete conversion to anatase titanium dioxide nanopowder of main particles size of 77 nm with major percentage of 70%. Chemical and physical characterizations were carried out for evaluation of the obtained products including transmission electron microscope, EDX, XRD, and the scanning advanced electronic diffraction pattern. Preliminary economic indicators were calculated to show that the capital cost of the plant is $1.613 million, with annual operating cost of $0.915 million which means the required investment is $2.528 million. The operating cost for the production of nanometric anatase TiO_2_ is $30.5/kg with depreciation excluding the land price.

## Introduction

Due to the excellent properties of titanium and their alloys, they have numerous applications and uses in many fields such as medical components, aerospace, chemical industries, automotive, sports and architecture (Diamanti et. al 2011, Leyens and Peters 2003, Brunette et al. 2001, Frazier et al. 2004, and Montgomery and Wells 2001). Also, titanium minerals are explored to produce titanium dioxide (TiO_2_) which is commonly used in cosmetics and food industries besides its uses as filler and white pigments in construction materials (Phillips and Barbano 1997, and Hewitt 1999). Any improvement in surface properties or generally in adaptation of physical techniques of TiO_2_ production leads to a significant increase in its scope of application. Main physical properties that specify the utility and uses of TiO_2_ in industrial applications are as follows: melting point, refractive index ant density (Varner 2010), gas sensing (Ramanavicius et al. 2022). Nano TiO_2_ has different applications than TiO_2_ because it is not inert but it is UV light attenuator on contrary to pigment (Woodrow Wilson International Center for Scholars 2010). The main two applications of nano TiO_2_ are to use it as UV attenuator or to use it as semiconductor or catalyst. Some applications of nano TiO_2_ are still under investigations in research and development phase and it is focused now on their conductive, photovoltaic, and catalytic properties (Yang et al. 2009a, b, Mahmoodi and Arami, 2009, and Labbe 2008). TiO_2_ can be synthesized by different methods such as gas phase and solution techniques (Ali et al. 2018). Several investigators focused on preparation of nano crystalline TiO_2_ with high surface area because it draws considerable attention as a photocatalyst (Mills and Le Hunte 1997). It is also projected to play an important role in solving pollution and environmental problems (Gratzel et al. 2003). TiO_2_ nanostructures can be synthesized by solution procedure or by gas phase procedure (Ali et al. 2018; Uon et al. 2020). Solution method can be divided to hydrothermal procedures (Yang et al. 2003), sol gel route (Vorkapic and Matsoukas 1998), and electrochemical procedures (Cargnello et al. 2014). Nanometric titanium dioxide (TiO_2_) is one of the most important ceramic materials that the whole world is currently interested in studying, because of its great industrial applications based on its physical and chemical properties such as its particle size, surface area, structural composition, thermal stability, and porosity (Yanting et al. 2009). Nanoscale titanium dioxide particles may be used as catalyst and photocatalyst, used in fabrication of solar panels, photovoltaic cells, and gas detection sensors (Congxue et al. 2008; Zheng et al. 2009). Tetragonal-anatase phase is the most important form of nanometric titanium dioxide among its three forms (anatase, brookite, and rutile); this is because of its excellent catalytic behavior (Nolph et al. 2007; Pedraza et al. 2009). There are many documented methods for preparing nanometric titanium oxide in its tetragonal-anatase structure, and these methods are limited to a sol–gel method, ultrasonic technology, micro-emulsion or reverse micellar method, hydrothermal processes, and finally the hydrolysis of inorganic salts (Kim et al. 2007, Lu and Wen 2008, Štengl et al. 2008, 2010). The most common techniques for anatase structure preparation are hydrothermal process and sol–gel method (Chuang et al. 2009; Collazzo et al. 2011). The most important raw material for nanometric titanium dioxide preparation is nano titanium hydroxide which is obtained using different polar and nonpolar solvent calcination of titanium hydroxide in temperature range of 200 to 600 °C which leads to the production of anatase structure nanometric titanium dioxide (Sivakumar et al. 2002; Akarsu et al. 2006). Some disadvantages of the sol–gel method and hydrothermal processes have been observed such as the high production cost and low reproducibility in sol gel while the hydrothermal method requires specialized equipment, high temperatures, and pressures for production (Chuang et al. 2009). Matijevic et al. (1977) prepared a titanium oxide sol precursor with very homogenous particle size distribution by means of aging a very acid solution of TiCl_4_ at 95 °C, using sulfate ions as control agent. Kato et al. (1989) synthesized spherical titanium oxide particles from an aqueous solution of TiOSO_4_ and urea as precipitating reagent, carrying out the material synthesis between 70 and 90 °C. Serpone et al. (1995) reported nanometric titanium oxide synthesis by controlled hydrolysis of TiCl_4_ at 0 °C, which led to obtain an oxide with anatase structure and uneven particle size. Park et al. (1996) reported thermal hydrolysis of Ti(SO_4_)_2_ at 80 °C in a mixture of 1-propanol and water, obtaining the anatase structure when the amorphous-hydrated precursor was calcined below 600 °C and rutile for calcination temperatures above 800 °C. Iwasaki et al. (1998) synthesized nanocrystalline titanium oxide with anatase structure by thermal hydrolysis of titanyl sulfate in a water/alcohol solution; adjusting the synthesis conditions such as water/alcohol ratio and reflux time, it was possible to obtain crystal sizes between 2 and 7 nm. Recently, Xu et al. (2008) synthesized titanium oxide micro-tubes modified with nitrogen from titanium tetra-chloride, using ammonium hydroxide as precipitant. The material obtained after calcination at 500 °C showed high thermal stability and good photocatalytic behavior in degradation of phenol and methyl orange, irradiating the test solution with visible and ultraviolet light. Kozlov et al. (2000) noted that titanium oxides synthesized from the hydrolysis of titanium tetrachloride have high surface acidity, which increased its catalytic activity in the photo-oxidation of ethanol in the gaseous phase. From the point of view of nano safety, it is recommended to produce any nanometric material in the range above of 30 nm. So, we are looking for eco-green production method of anatase nanometric titanium dioxide. One of the most promising methods is the anodic dissolution of titanium substrate in saline electrolyte solution.

### Titanium dioxide applications

It is clear that the various preparation procedures have a significant impact on the microstructure of TiO_2_, such as its shape and particle size. Some TiO_2_ properties are dramatically altered by the various processes, resulting in a variety of applications. TiO_2_ is found in three crystal structures: anatase, rutile, and brookite. The crystalline structure of anatase-phased TiO_2_ is established in the tetragonal system with bipyramidal system. This substance is used in cosmetics, particularly for sun protection, while rutile-phased TiO_2_ has a crystalline structure that is similar to the tetragonal system with a prismatic system. Rutile is used in the production of paint, polymers, coatings, and cosmetics. The crystalline structure of brookite-phased TiO_2_ features an orthorhombic system. These crystalline polymorphic forms make it appropriate for a variety of technical applications due to its chemical stability and low toxicity (Buraso et al. 2018, Gupta and Tripathi 2012, Viana et al. 2010, Ramanavicius et al. 2019, 2020). TiO_2_ nanoparticles have been manufactured using a variety of processes, including the sol–gel method, aerosol process, the inert gas condensation, and the hydrothermal process. Many procedures are involved in the producing using the Sol–gel approach. In certain circumstances, it also employs expensive chemicals (Asep et al. 2022, Zhang et al. 2002, Gupta and Tripathi 2012, Viana et al. 2010), while a simple approach is used to manufacture high-purity TiO_2_ nanoparticles in the aerosol process. However, the high temperature used in this technique causes issues with manufacturing costs. Because of the usage of specialized equipment such as ultrahigh vacuum, the inert gas condensation method has a high manufacturing cost. The hydrothermal process may generate particles at low temperatures (less than 300 °C) (Suryanarayana and Prabhu 2006, Zhang et al. 2002, Gupta and Tripathi 2012).

## Experimental

### Apparatus

**Fig. 1:**
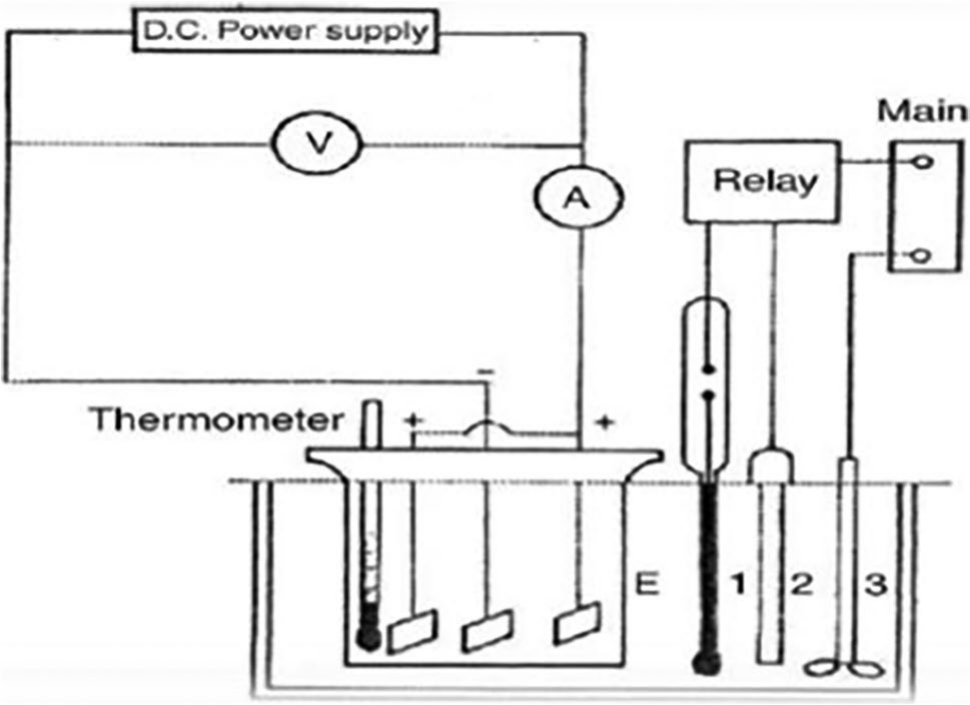
Electrolysis cell used for synthesis of nano titanium hydroxide

Figure 1 shows the electrochemical cell where titanium substrate is anodically dissolved to generate titanium hydroxide powder.

### Material and methods

#### Materials

Nitric and sulfuric acid were obtained from El-Nasr Pharmaceutical Chemical Co., pure reagents for analysis. Titanium rods of high analytical grade were fixed as anode and cathodes manufactured by El Naser Co. for chemicals.

#### Procedures

Anodic dissolution of titanium substrate .

Figure 1 shows the electrochemical cell used for titanium rods anodic dissolution and generation of nano titanium hydroxide powder. The cell is a 250-ml capacity Plexi-glass rectangular cell. Three high analytical grade titanium rods of external area of 20-cm^2^ dimensions were used as one anode which is hung in the middle pathway of two titanium cathodes. D.C. power supply of 50 V and 5A with digital display, voltage, and current control with sensitivity of 0.1 V and 0.1A was used for supplying of direct electric current. Temperature controller was used for adjustment of the electrolysis process temperature within range of 20 to 100 °C. The electrolyte solution is stirred during the electrolysis process using magnetic stirrer within 0–500 rpm. Low concentration sodium chloride solution (3 g/l) was used as electrolyte for titanium anodic dissolution process for generation of nano titanium hydroxide particles. The rods were polished via sand paper and soaked in acetone for 3 min; after that, they were subjected for alkaline degreasing to remove oils and fats adhered at their surfaces. The alkaline solution containing 0.5 M NaOH, 0.5 M Na_2_CO_3_, and 10 g/l EDTA was used for degreasing by soaking for 15 min at 50 °C. The degreased titanium rod was rinsed with running water then acidified by 0.1 M sulfuric acid. Once the electrodes submerged in the sodium chloride solution, D.C. current is adjusted at the desired value with stirring. At the end of the electrolysis time, the solution constitutes the generated nano-metric titanium hydroxide particles that is lifted for about 24-h filtering followed by rinsing with running water carried out until the pH reached 7. The mixture was filtered and dried and the resulting titanium hydroxide was ground. Operating conditions such as effect of electrolyte type, solution pH, applied current density, electrolysis time, solution temperature, electrode gap distance, and sodium chloride concentration were investigated for the preparation of nanometric titanium hydroxide powder. The synthesized titanium hydroxide powder is calcined at temperature range of 400 to 800 °C for 6 h to be converted to nanometric titanium dioxide powder. Both the effect of calcination temperature and calcination time was studied for the investigation of the optimum conditions for conversion of nanometric titanium hydroxide to nanometric titanium dioxide powder. The generated nano titanium dioxide particles were tested for adsorption of cationic dyes for instance methylene blue dye solution of 100 mg/l. Both of the obtained nanometric titanium hydroxide and nanometric titanium dioxide particles were subjected to the analytical and physical characterization.

### Characterization

#### XRD diffraction

X-ray diffraction (XRD) was carried out using Bruker’s D8 advanced X-ray diffract meter via CuKα radiation (λ = 1.5418 °A). Dynamic light scattering (Model no: HORIBA and nano particle analyzer SZ100) was used to measure the particle size. XRD was conducted at a scanning speed of 1 s/step, scanning range of 3–70° (2θ), and resolution of 0.05°/step. The used electrolysis cell for synthesis of nano titanium hydroxide is shown in Fig. 1

#### Transmission electron microscope test

The obtained nano-particles were characterized by means of a JEOL-JEM1200 transmission electron microscope (TEM). The TEM sample was prepared by adding a drop of the nano-solution on a 400-mesh copper grid coated by an amorphous carbon film and let the sample to dry in open air at room temperature. The average diameter of nano-particles was determined within the range of 100 nm that was found in several chosen areas in enlarged microphotographs. Moreover, the same electron microscope JEOL-JEM-1200 was used for the examination of the selected area electron diffraction patterns tested particles.

#### SEM–EDX analysis

The surface morphology of the nano titanium hydroxide and nano titanium dioxide particles were investigated via SEM–EDX analysis.

#### Evaluation of nano Ti(OH)_4_ to nano TiO_2_ conversion

Based on the dissolution behavior of both titanium hydroxide and titanium dioxide, separation process and determination of titanium ion in both compounds were carried out. Titanium hydroxide is soluble in both diluted strong and weak acids, while titanium dioxide is soluble in hot strong concentrated acids. So, the generated titanium dioxide after calcination is subjected in diluted hydrochloric acid (0.1 M) and stirred for 60 min, then filtered to give solution 1 (Ti_H_) and the precipitates which is then dissolved in hot concentrated hydrochloric acid (3 M) to give solution 2 (Ti_O_), while the dissolution of the calcined sample will give solution 3 which is total titanium (Ti_T_), and both of the obtained clear solutions are then subjected for measurement using atomic absorption as described by Shokrollahi and Gohari (2016). The conversion efficiency of nano titanium hydroxide to nano titanium dioxide is calculated from the following equation:

1 $$\mathrm{Conversion}\;\mathrm{Efficiency}\;(\%)={}^{(\mathrm{TiT})}/_{\mathrm{TiH}}\times100$$

## Results and discussion

The preparation of nanometric titanium dioxide through the anodic dissolution of titanium rod have to pass through the generation of nanometric titanium hydroxide powder which will be then converted to nanometric titanium dioxide using heat treatment. Several operating conditions controlling the anodic dissolution of titanium rod and the generation of nanometric titanium hydroxide powder such as electrolyte pH, applied current density, electrolysis time, solution temperature, sodium chloride concentration, and electrode gap distance were investigated. In the following part, investigation of the optimum conditions for the generation of nanometric titanium hydroxide powder was carried out.

### Effect of electrolyte type

**Fig. 2:**
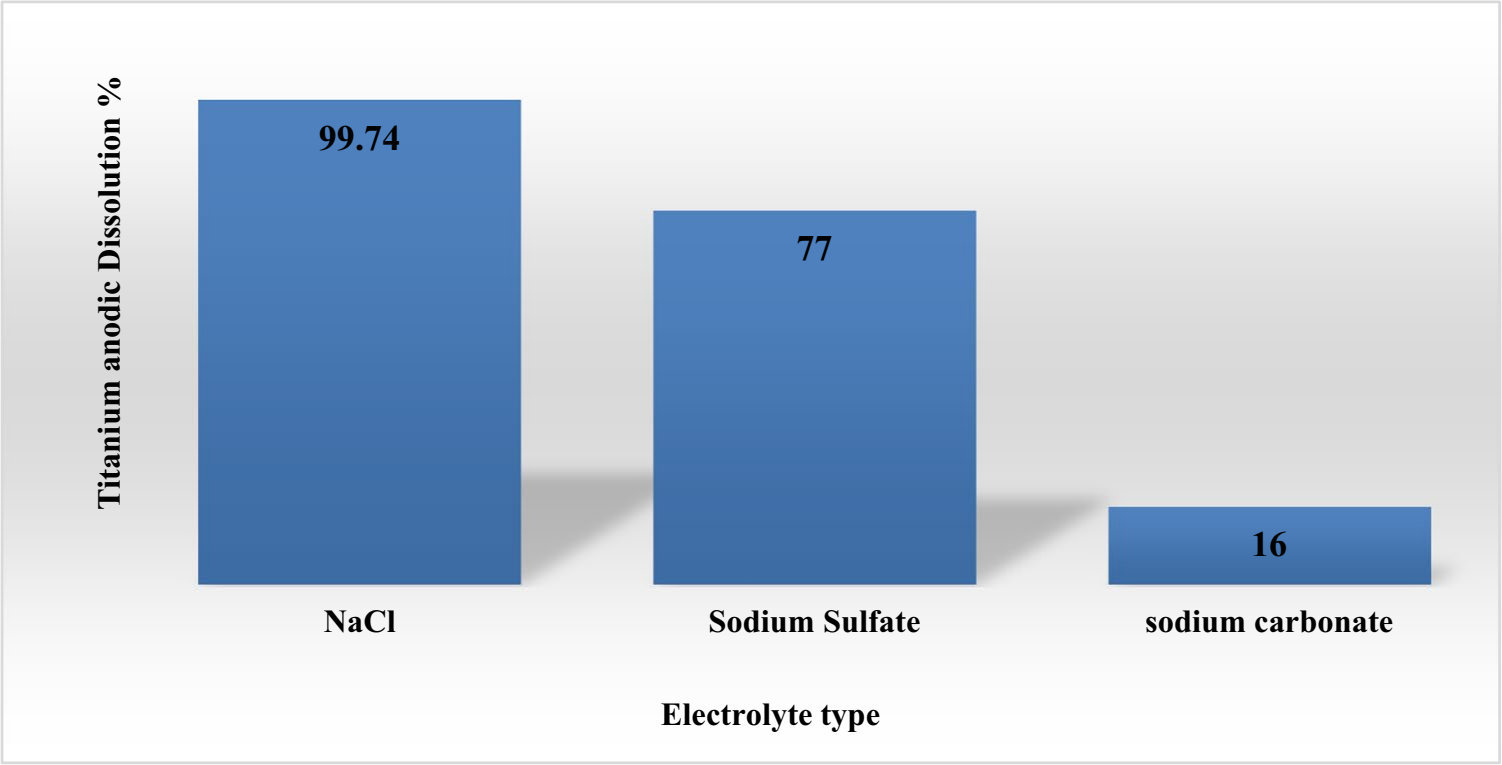
Effect of electrolyte type on anodic dissolution efficiency of titanium substrate for generation of titanium hydroxide at operating conditions: C.D. 75 mA/cm^2^, 25 °C, 150 rpm, electrode gap distance 3 cm, and electrolyte concentration 3 g/l for electrolysis time 120 min

Figure 2 shows the effect of electrolyte type used for anodic dissolution of titanium substrate to generate nano titanium hydroxide at electrolysis conditions: pH 4, C.D 75 mA/cm^2^, electrolysis time 120 min, electrolyte concentration 3gm/l, electrode gap distance 3 cm, stirring rate 150 rpm at electrolysis temperature 20 °C. The results indicate that sodium chloride gives the maximum anodic dissolution efficiency of titanium substrate which is 99.74%, while it was 77% for sodium sulfate and 16% for sodium carbonate, respectively. So, sodium chloride was selected as the optimum electrolyte for the generation of nano titanium hydroxide via anodic dissolution of titanium metal.

### Effect of pH

**Fig. 3:**
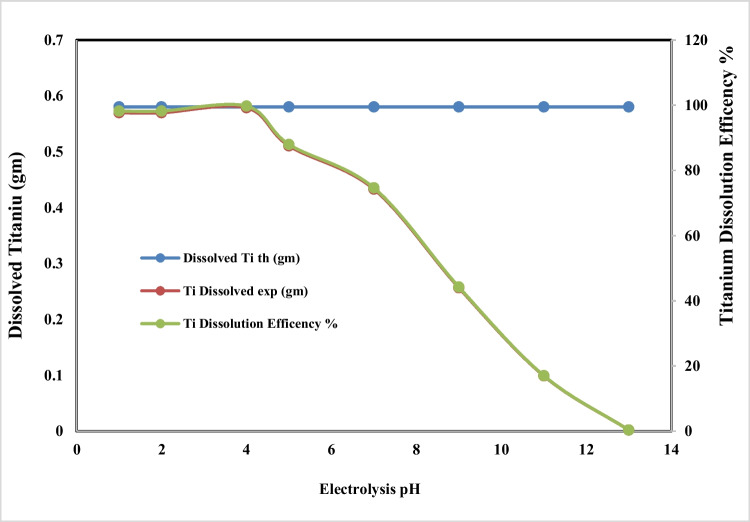
Effect of electrolysis pH on dissolved titanium and Faraday’s dissolution efficiency during titanium metal anodic dissolution at operating conditions: C.D. 65 mA/cm^2^, 25 °C, 150 rpm, electrode gap distance 3 cm, and NaCl 3 g/l for electrolysis time 120 min

**Fig. 4:**
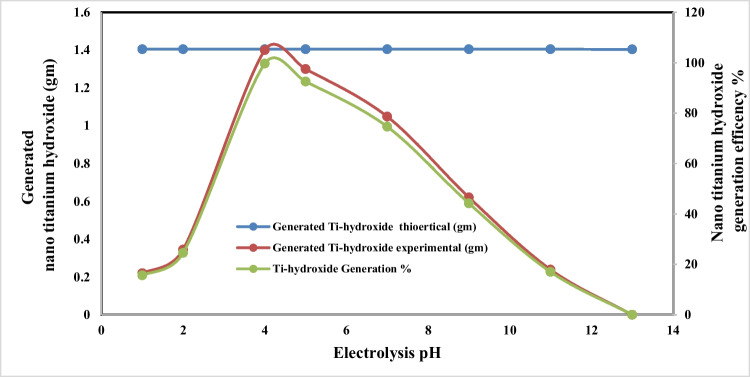
Effect of electrolysis pH on generated nano titanium hydroxide weight and its formation efficiency during titanium metal anodic dissolution at operating conditions: C.D. 65 mA/cm^2^, 25 °C, 150 rpm, electrode gap distance 3 cm, and NaCl 3 g/l for electrolysis time 120 min

For studding the effect of electrolyte pH on the anodic dissolution of titanium rod, the other operating conditions were fixed at applied current density of 75 mA/cm^2^, electrolysis time 30 min, sodium chloride concentration of 3gm/l, electrode gap distance 3 cm, and stirring rate 150 rpm at 20 °C. Figures 3 and 4 show the effect of the pH on the titanium metal dissolution (weight and efficiency) and the generation efficiency of nano titanium hydroxide powder, respectively. It is clear from Fig. 4 that, as the pH increased from 1 to 4, the dissolved titanium is about 0.5804gm and the Faradays dissolution efficiency is within the range of 97.9%. After that, as the pH increased, the dissolved titanium decreased and also the dissolution efficiency which reached to zero at pH 13; this may be attributed to the electrode passivation within this pH range from 6 to 13. On the other hand, at low pH, the dissolved titanium conversion efficiency to nanometric titanium hydroxide is low within the range of pH 2–4 which may be attributed to the strong acidic medium which keeps the dissolved titanium as titanium chloride. As the pH increases, the titanium hydroxide generation efficiency increases reaching its maximum value at pH 4 which is about 99% then it started to decrease again due to the decreasing of the dissolved titanium due to electrode passivation as clear in Fig. 3. Based on the above, pH value of 4 was taken as optimum.

### Effect of applied current density

**Fig. 5:**
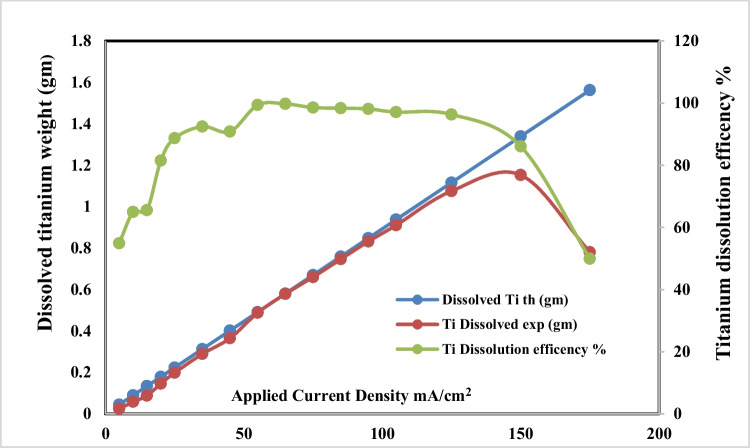
Effect of applied current on dissolved titanium metal weight and Faraday’s dissolution efficiency during titanium substrate anodic dissolution at operating conditions: pH4, 25 °C, 150 rpm, gap distance 3 cm, and NaCl 3 g/l for electrolysis time 120 min

**Fig. 6:**
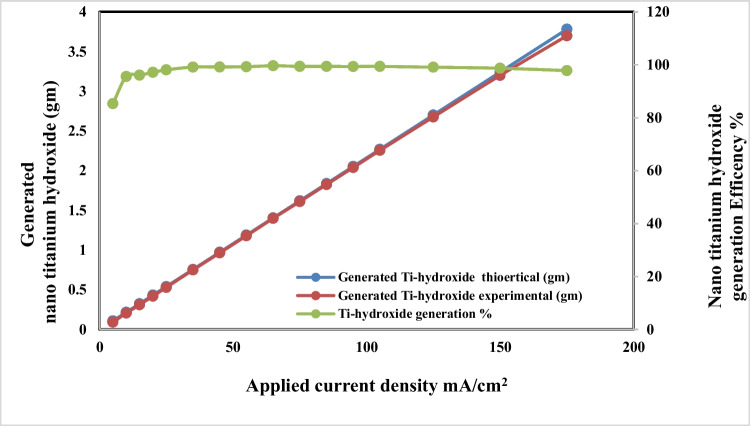
Effect of applied current on generated nano titanium hydroxide weight and its formation efficiency during titanium substrate anodic dissolution at operating conditions: pH4, 25 °C, 150 rpm, gap distance 3 cm, and NaCl 3 g/l for electrolysis time 120 min

The effects of applied current density on dissolved titanium, anodic dissolution current efficiency, and conversion of dissolved titanium to nanometric titanium hydroxide powder are respectively graphically represented in Figs. 5 and 6 during the titanium anodic dissolution process. These were experimentally carried out at pH 4, sodium chloride concentration 3 g/l, electrolysis time 120 min, and electrode gap distances 3 cm, with stirring rate 150 rpm at 25 °C. The results indicate that as current density increases, the dissolved titanium ions generated increases too, and the formed titanium hydroxide increases also until current density 65 mA/cm^2^, after that although current density increases, generated titanium hydroxide efficiency is decreased; this may be attributed to the formation of adhered black powder layer at the electrode surfaces during dissolution which decreases the dissolved titanium. Based on the above results, applied current density of 65 mA/cm^2^ was taken as optimum.

### Effect of electrolysis time

**Fig. 7:**
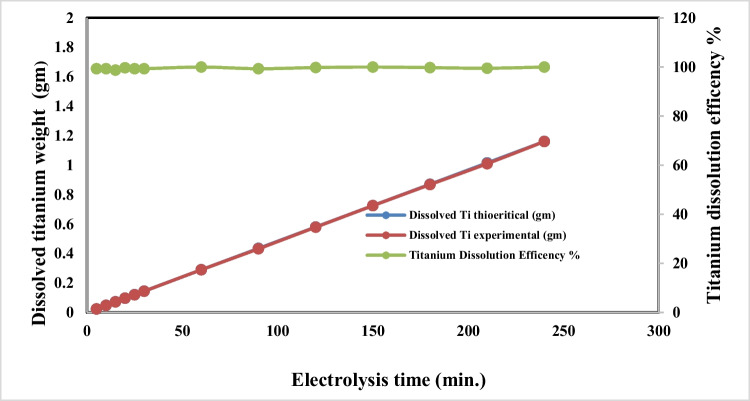
Effect of electrolysis time on dissolved titanium metal weight and Faraday’s dissolution efficiency during titanium substrate anodic dissolution at operating conditions: pH4, C.D. 65 mA/cm^2^, 25 °C, 150 rpm, NaCl 3 m/l, and electrode gap distance 3 cm

**Fig. 8:**
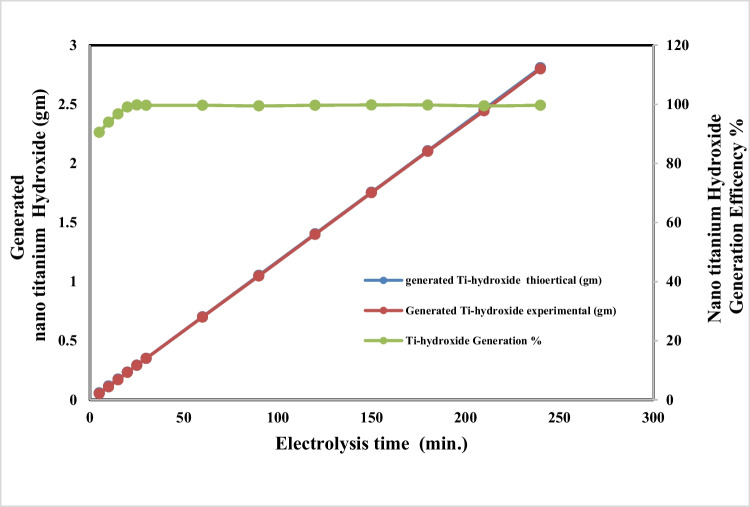
Effect of electrolysis time on generated nano titanium hydroxide and its formation efficiency during titanium substrate anodic dissolution at operating conditions: pH4, C.D. 65 mA/cm^2^, 25 °C, 150 rpm, NaCl 3 m/l, and electrode gap distance 3 cm

Figures 7 and 8 represent the effect of electrolysis time on both titanium anodic dissolution process and titanium ion conversion to nanometric titanium hydroxide, respectively, at applied current density 65 mA/cm^2^, pH 4, and sodium chloride concentration 3 g/l, electrode gap distance of 3 cm, and stirring rate 150 rpm at 25** °C**.

The results show that as the electrolysis time increases, the generated dissolved titanium ions and the formed nanometric titanium hydroxide increase, while both the anodic dissolution current efficiency and conversion to nano titanium hydroxide efficiency have constant values without any observation of changes in the titanium anode surfaces. Based on the above, 240 min was taken as optimum electrolysis time for titanium anodic dissolution process, to maximize the generated nanometric titanium hydroxide powder weight.

### Effect of sodium chloride concentration

**Fig. 9:**
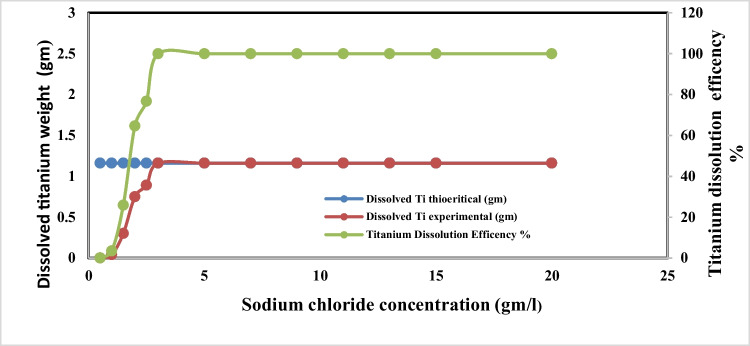
Effect of sodium chloride concentration on dissolved titanium metal weight and Faraday’s dissolution efficiency during titanium substrate anodic dissolution at operating conditions: pH4, C.D. 65 mA/cm^2^, 25 °C, 150 rpm, and gap distance 3 cm for electrolysis time 240 min

**Fig. 10:**
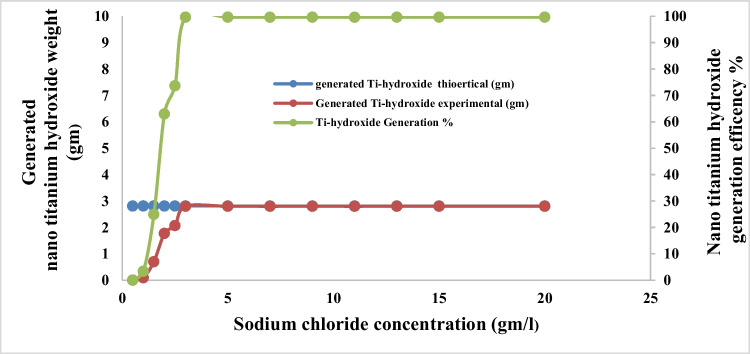
Effect of sodium chloride concentration on generated nano titanium hydroxide weight and its formation efficiency during titanium substrate anodic dissolution at operating conditions: pH4, C.D. 65 mA/cm^2^, 25 °C, 150 rpm, and gap distance 3 cm for electrolysis time 240 min

Figures 9 and 10 show the effect of sodium chloride concentration on dissolved titanium weight, anodic dissolution current efficiency, and the conversion of generated dissolved titanium ion to nanometric titanium hydroxide powder, respectively, during the anodic dissolution process, at the following operating conditions: pH 4, applied current density of 65 mA/cm^2^, electrolysis time 240 min, electrode gap distance 3 cm, with stirring of 150 rpm, at 25 °C.

The results indicated that, with increasing sodium chloride concentration, the generated dissolved titanium weight, the anodic dissolution current efficiency, and the conversion of the generated dissolved titanium ion to nanometric titanium hydroxide powder increase reaching their maximum values at sodium chloride concentration 3 g/l then no significance change obtained at higher concentration of sodium chloride. So 3 g/l sodium chloride was taken as optimum electrolyte concentration during titanium anodic dissolution process.

### Effect of temperature

**Fig. 11:**
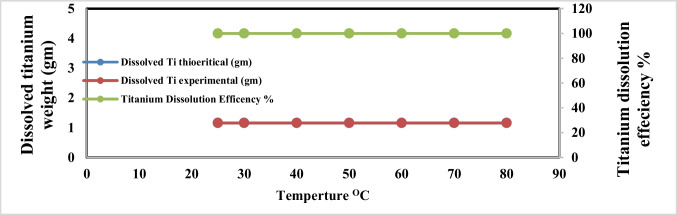
Effect of electrolyte temperature on dissolved titanium metal weight and Faraday’s dissolution efficiency during titanium substrate anodic dissolution at operating conditions: pH4, C.D. 65 mA/cm^2^, NaCl 3 g/l, 150 rpm, and gap distance 3 cm for electrolysis time 240 min

**Fig. 12:**
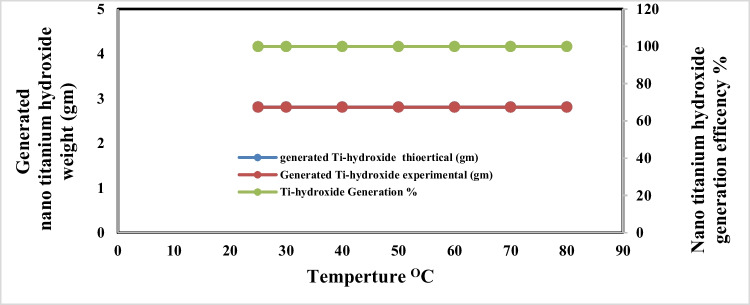
Effect of electrolyte temperature on generated nano titanium hydroxide weight and its formation efficiency during titanium substrate anodic dissolution at operating conditions: pH4, C.D. 65 mA/cm^2^, NaCl 3 g/l, 150 rpm, and gap distance 3 cm for electrolysis time 240 min

The effect of electrolyte temperature changes during anodic dissolution of titanium metal in sodium chloride solution at operating conditions pH 4, C.D. 65 mA/cm^2^, and electrolysis time 240 min, NaCl 3 g/l, electrode gap distance 3 cm, and 150 rpm was studied. Figures 11 and 12 show that there is no effect of the electrolyte temperature increment on the titanium metal anodic dissolution efficiency and generation efficiency of nanometric titanium hydroxide efficiency, so from the economic point of view, 25 °C was taken as optimum.

### Effect of electrode gap distance

**Fig. 13:**
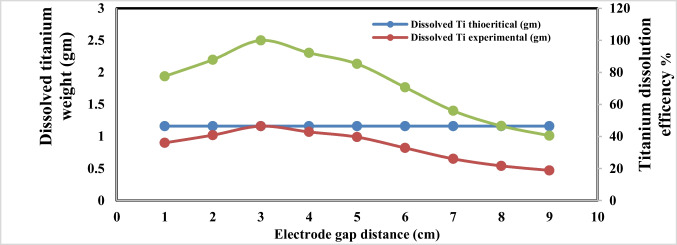
Effect of electrode gap distance on dissolved titanium metal weight and Faraday’s dissolution efficiency during titanium substrate anodic dissolution at operating conditions: pH4, C.D. 65 mA/cm^2^, 25 °C, 150 rpm, and sodium chloride 3 g/l for electrolysis time 240 min

**Fig. 14:**
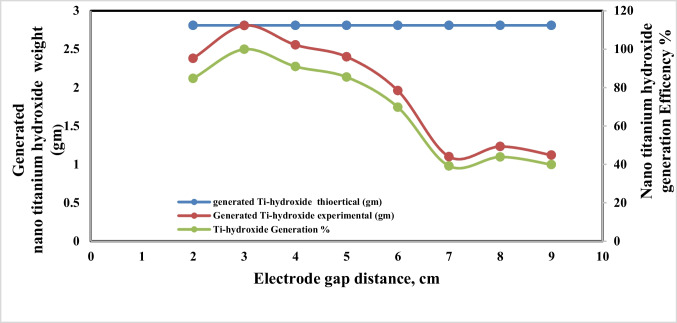
Effect of electrode gap distance on generated nano titanium hydroxide weight and its formation efficiency during titanium substrate anodic dissolution at operating conditions: pH4, C.D. 65 mA/cm^2^, 250 °C, 150 rpm, and sodium chloride 3 g/l for electrolysis time 240 min

**Fig. 15:**
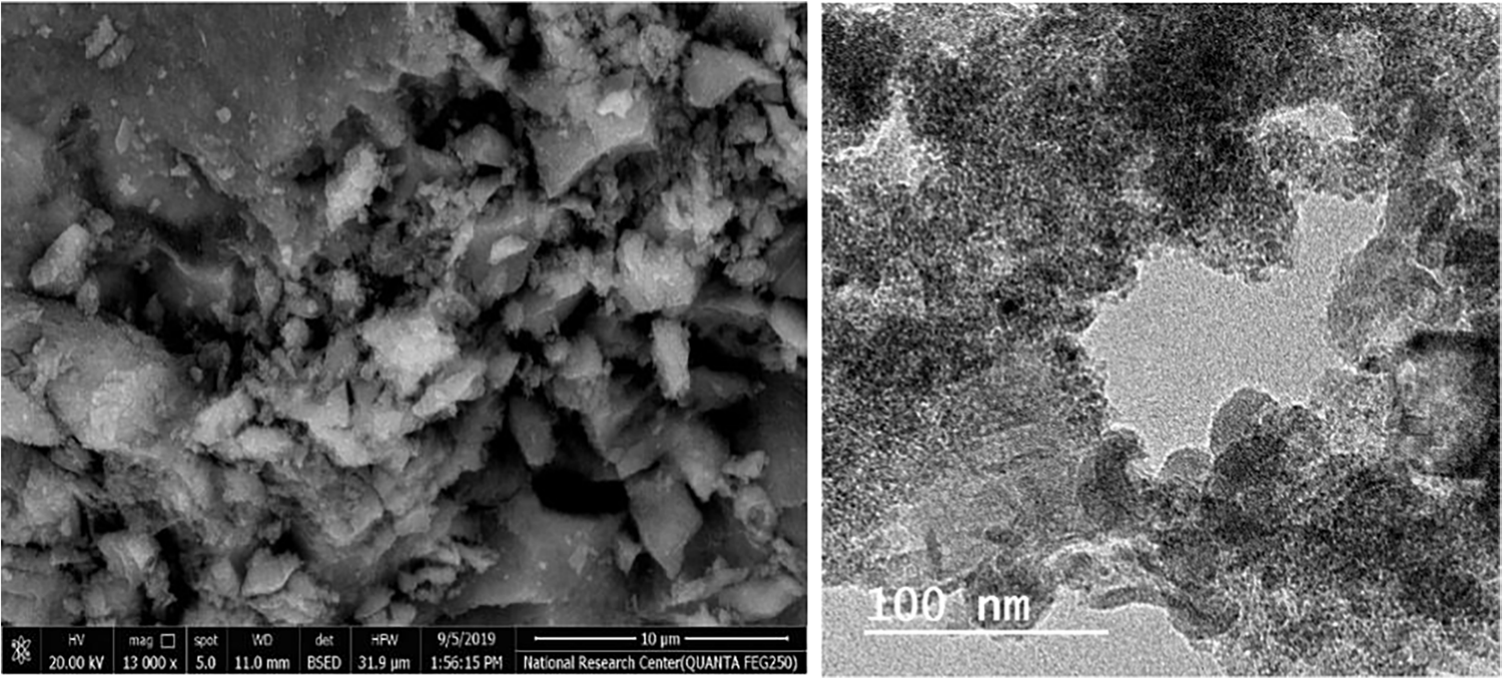
the SEM and TEM analysis of obtained nano titanium hydroxide generated via anodic dissolution of titanium metal at the optimum selected electrolysis conditions of: pH 4, C.D. 65 mA/cm^2^, 25 °C, 150 rpm, electrode gap distance 3 cm, and NaCl 3 g/l for electrolysis time 120 min

**Fig. 16:**
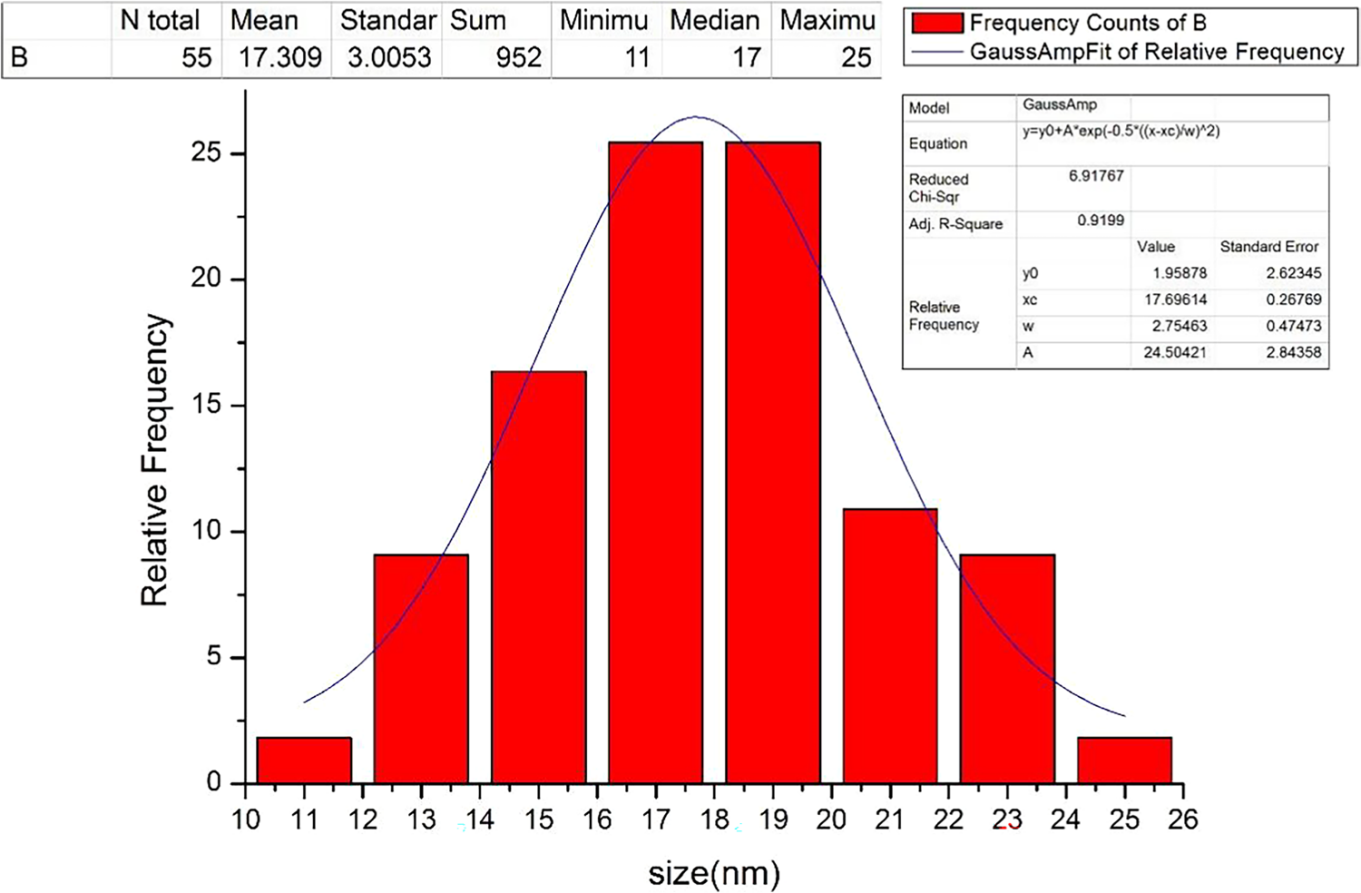
the histogram of obtained nano titanium hydroxide generated via anodic dissolution of Titanium metal at the optimum selected electrolysis conditions of pH 4, C.D. 65 mA/cm^2^, 25 °C, 150 rpm, electrode gap distance 3 cm, and NaCl 3 g/l for electrolysis time 120 min

Figures 13 and 14 indicate that at the operating conditions pH 4, electrolysis time 240 min, sodium chloride concentration 3 g/l, C.D. 65 mA/cm^2^, and 150 rpm at 25 °C, the results show, as the gap distance decreased less than 3 cm, no significance change occurred but the observations shows that black powder is obtained within the light white nanometric titanium hydroxide powder; this may be attributed to the high attack of the electric current and the exceeding of the titanium oxidation potential, while at electrode gap distance more than 3 cm there are decreasing in the dissolved titanium, obtained nanometric titanium hydroxide powder, anodic dissolution efficiency, and nanometric titanium hydroxide generation efficiency; these may be due to the decreasing of the throwing power between electrodes, so 3 cm as electrode gap distance may be taken as optimum value. Based on the above, the optimum electrolysis conditions for anodic dissolution of titanium substrate for generation of nano titanium hydroxide were selected as pH 4, C.D. 65 mA/cm^2^, 25 °C, 150 rpm, electrode gap distance 3 cm, and NaCl 3 g/l for electrolysis time 120 min. At these operating conditions, the dissolved titanium, generated nano titanium hydroxide nanometric particles, titanium dissolution efficiency, and titanium hydroxide nanometric generation efficiency were 1.16 g, 2.8067 g, 99.938%, and 99.899%, respectively. The nanometric titanium hydroxide particles generated sample was subjected for SEM and TEM analysis as shown in Fig. 15. Scanning electron microscopy (SEM) was used for morphological studies. SEM images show the thin nanoparticles of titanium hydroxide generated from anode dissolution of titanium metal using electrochemical decomposition at magnification (2000kv). In addition, from the atomic TEM measurements, that shows the generated nanometric titanium hydroxide particles of square-shaped flacks with dimensions ranging from 11 to 25 nm. The histogram of the obtained nanometric titanium hydroxide particles is graphically presented in Fig. 16.

### Effect of calcination temperature

**Fig. 17:**
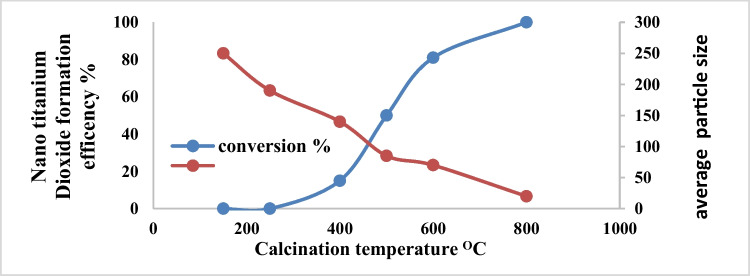
Effect of calcination temperature on the conversion efficiency of nanometric titanium hydroxide to nano metric titanium dioxide at 3 h calcination time

Twenty-five grams of the electro-generated nanometric titanium hydroxide at optimum electrolysis conditions as mentioned above was subjected to calcination for 3 h for conversion of the nanometric titanium hydroxide to nanometric titanium dioxide. The most important operating parameters for this process are calcination temperature and calcination time. Figure 17 shows the effect of the calcination temperature on the obtained nanometric titanium dioxide. As it is clear from Fig. 17 that as the calcination temperature increases, the obtained particle sizes decrease from average particle size of 250 nm reaching to average particle size of 20 nm at calcination temperature of 800 °C. From the point of view of nano safety, 20 nm cannot be taken as optimum and also this very small particle size may be attributed to the formation of nano metric titanium oxide not titanium dioxide nanometric particles. From the point of view of weight loss, the maximum weight loss is obtained at 800 °C. The target nanometric size is in the range of 50 to 80 nm which is obtained in calcination temperature of 500 °C, and 600 °C, with conversion efficiency of 50%, and 81%, respectively. Based on the above, calcination temperature of 600 °C is taken as optimum temperature for the conversion of nanometric titanium hydroxide to nanometric titanium dioxide. It is clear from Fig. 17 that, as the calcination temperature increases, the conversion efficiency of nano titanium hydroxide to nano titanium oxide increases and the obtained nano size decreases reaching to 81% and 70 nm, and 100% and 20 nm, at 600 °C and 800 °C respectively. From the point of nano-safety and the energy saving with economic considerations, 600 °C was taken as optimum calcination temperature for the conversion of nano titanium hydroxide to nano titanium oxide.

### Effect of calcination time

**Fig. 18:**
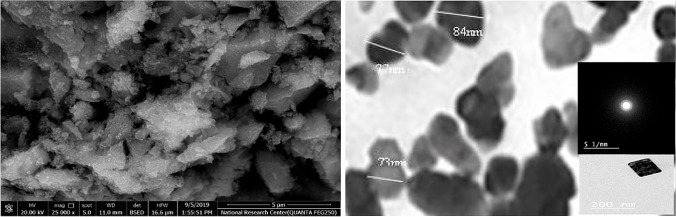
SEM, TEM, and SAED analysis of the generated nanometric titanium dioxide particles obtained via calcination at (600 °C and 240 min) of nanometric titanium hydroxide electrogenerated via anodic dissolution of titanium metal at pH 4, C.D. 65 mA/cm^2^, 25 °C, 150 rpm, electrode gap distance 3 cm, and NaCl 3 g/l for electrolysis time 120 min

**Fig. 19:**
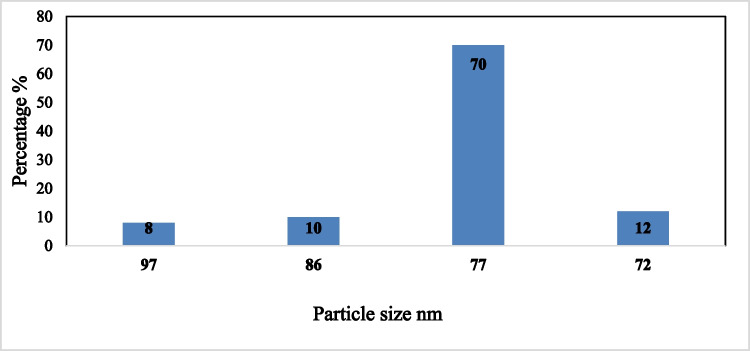
the histogram of obtained nano titanium dioxide generated via anodic dissolution of Titanium metal at the optimum selected electrolysis conditions of: pH 4, C.D. 65 mA/cm^2^, 25 °C, 150 rpm, electrode gap distance 3 cm, and NaCl 3 g/l for electrolysis time 120 min, then calcinated at 600 °C for 240 min

Figure 18 shows the SEM, TEM, and scanning advanced electronic diffraction (SAED) analysis of the obtained titanium dioxide nano metric particles obtained by the anodic dissolution of titanium metal in saline solution for generation of nanometric titanium hydroxide powder followed by the calcination at 600 °C and 240 min for complete conversion to nanometric titanium dioxide particles. The SEM analysis shows homogenous grains of titanium particles with sharp boundaries, the TEM analysis shows that the main particle size of the obtained nanometric particles is 77 nm, while the SAED analysis show that the electro dissolved metal is titanium metal. Figure 19 shows the histogram of the obtained nanometric titanium dioxide particles which exhibit the particle size distribution percentage through the obtained sample.

**Fig. 20:**
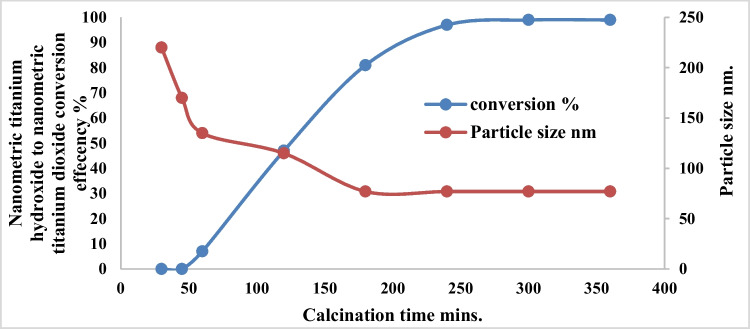
Effect of calcination time on the conversion efficiency of nanometric titanium hydroxide to nano metric titanium dioxide at 600 °C calcination temperature

**Fig. 21:**
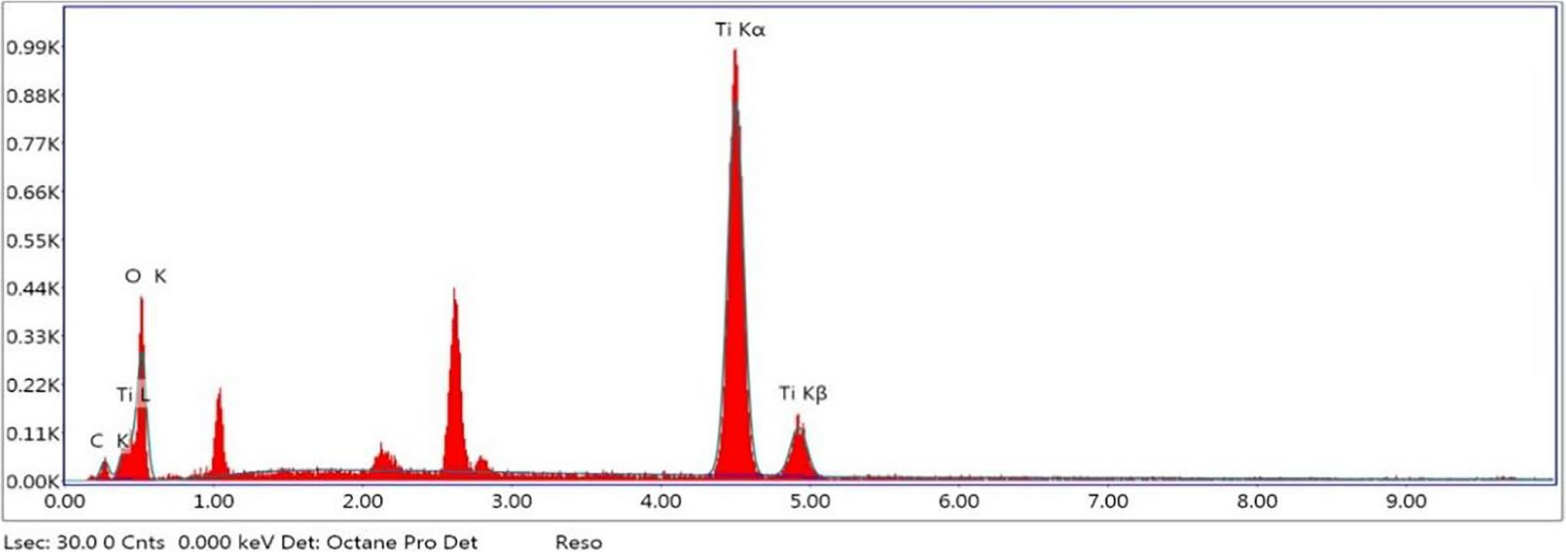
The energy-dispersive X-ray (EDX) of the generated nanometric titanium dioxide particles obtained via calcination at (600 °C and 240 min) of nanometric titanium hydroxide electrogenerated via anodic dissolution of titanium metal at pH 4, C.D. 65 mA/cm^2^, 25 °C, 150 rpm, electrode gap distance 3 cm, and NaCl 3 g/l for electrolysis time 120 min

Figure 20 shows the effect of calcination time on the conversion efficiency of nano titanium hydroxide to nano titanium dioxide and obtained nano size of titanium dioxide at calcination temperature of 600 °C. The results show that complete conversion obtained at 240 min with nano titanium dioxide seize of 77 nm. Based on the above results, it is clear that the production of about 65 to 80 nm anatase nanometric titanium dioxide powder via anodic dissolution of titanium metal required operating conditions of as pH 4, C.D. 65 mA/cm^2^, 25 °C, 150 rpm, electrode gap distance 3 cm, and NaCl 3 g/l for electrolysis time 120 min. These operating conditions lead to the formation of nanometric titanium hydroxide particles of main particle size of 250 nm which when calcinated at 600 °C for 240 min is completely converted to anatase nanometric titanium dioxide particles. This sample obtained at these optimum operating conditions is further subjected for more characterization such as energy-dispersive X-ray (EDX), XRD, TEM, SAED analysis, and finally the histogram of the generated nanometric titanium dioxide powder. Figure 21 shows the EDX results. All of the grown particles at the varied growth temperature and fixed baking time were proceeded with EDX analysis to determine the elemental composition. The results revealed the atomic percentage of titanium and oxygen of the grown particles. It can be concluded that all of them had the right atomic ratio of titanium to oxygen which is around 1:2.

**Fig. 22:**
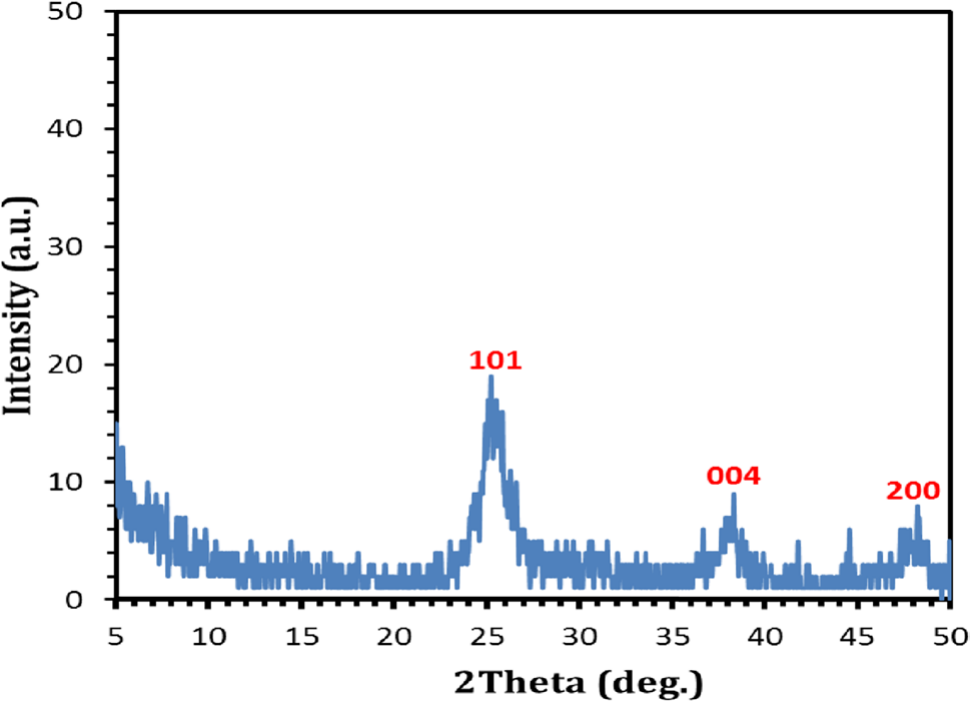
XRD of the generated nanometric titanium dioxide particles obtained via calcination at (600 °C and 240 min) of nanometric titanium hydroxide electrogenerated via anodic dissolution of titanium metal at pH 4, C.D. 65 mA/cm^2^, 25 °C, 150 rpm, electrode gap distance 3 cm, and NaCl 3 g/l for electrolysis time 120 min

The XRD was used to investigate the crystal structure of TiO_2_ nanoparticle. These patterns of TiO_2_ nanoparticle have some peaks shown in Fig. 22. The peaks occur at 2θ of 25.3° for crystal plane 101and 37.90 for crystal plane (004). The result shows that the crystal structure of the TiO_2_ is anatase and the interlayer distance of this compound was calculated (*d* spacing = 3.52732 Å, and 2.34287 Å respectively); these data agree with Bagheri et al. (2013) and Ghorbanpour et al. (2015)

2 $$\mathrm{Ti}-4\mathrm{e}\to {\mathrm{Ti}}^{+4}+4\mathrm{e}$$

3 $${\mathrm H}_2\mathrm O\rightarrow\mathrm{OH}^-+\mathrm H^+$$

4 $${\mathrm{Ti}}^{+4}+4{\mathrm{OH}}^{-}\to \mathrm{Ti}{\left(\mathrm{OH}\right)}_{4}$$

The overall reactions are:

5 $$\mathrm{Ti}+{4\mathrm{H}}_{2}\mathrm{O}\to \mathrm{Ti}{\left(\mathrm{OH}\right)}_{4}+2{\mathrm{H}}_{2}$$

6 $$\mathrm{Ti}{\left(\mathrm{OH}\right)}_{4}\to {\mathrm{TiO}}_{2}+2{\mathrm{H}}_{2}\mathrm{O}$$

## Preliminary financial indicators

**Fig. 23:**
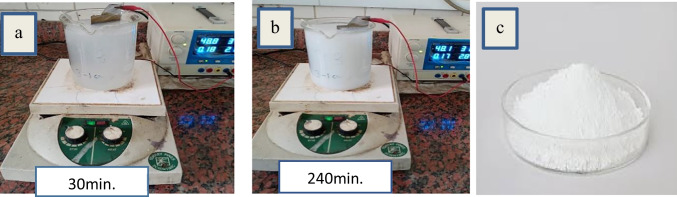
**a** The electrogenerated nanometric titanium hydroxide after 30 min electrolysis time, **b** the electrogenerated nanometric titanium hydroxide after 240 min electrolysis time, and **c** the obtained nanometric titanium dioxide after calcination at 600 °C and 240 min calcination time

**Fig. 24:**
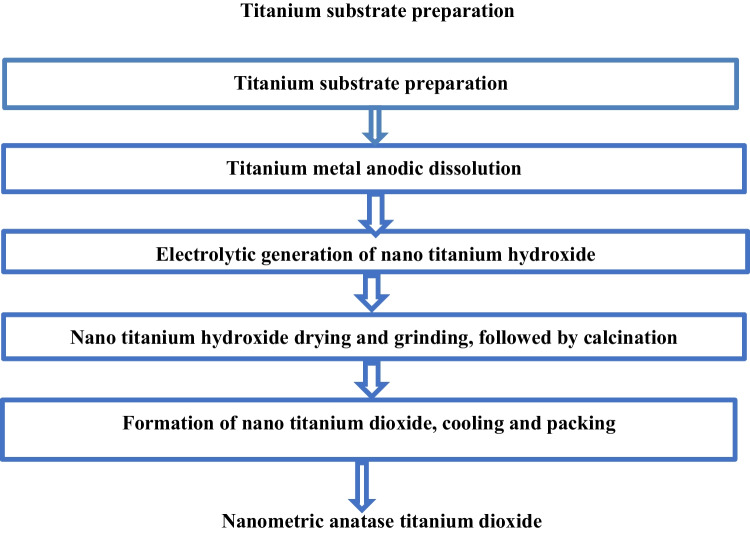
Block flow diagram for the green electrolytic production of nano titanium hydroxide and nanometric anatase titanium dioxide powder via anodic dissolution of titanium metal flowed by calcination at 600 °C for 4 h

Based on the lab-scale obtained results for the preparation of nanometric anatase titanium dioxide powder via anodic dissolution of titanium metal for generation of nano titanium hydroxide at operating conditions of pH 4, C.D. 65mA/cm^2^, 240min, 3g/l sodium chloride solution, electrode gap distance, and stirring rate 150rpm at 25°C, followed by calcination at 600°C for 240min, a preliminary design of production plant of 100kg/day production capacity with its economic indicators was carried out. Raw titanium ores are presently worth between $0.09 and $0.51 per kilogram. Processing them into bulk TiO_2_ adds an order of magnitude of value, since it sells for around $2.21/kg (Zucker and Darby 2004, Sun et al. 2014). Manufacturing nano-TiO_2_, which is still a specialized chemical, raises the value by two orders of magnitude; nano-TiO_2_ is sold for $176 to $198/kg, according to corporate queries (Zucker and Darby 2004, Sun et al. 2014). Technologically, the materials utilized and the production of TiO_2_ nanoparticles may be enhanced due to the ability of the industrial instruments to process huge numbers. It takes roughly one reaction cycle to create 100 kg of TiO_2_ nanoparticles per day. In this study, a novel cost-effective eco green electro-generation technology for production of nanometric titanium hydroxide and nanometric anatase titanium dioxide was described according to Fig. 23 a and b. In the materials used based on mechanism described by Eqs. 2 and 3, it takes on reaction cycle the usage of approximately 63 kg of titanium metal sheets, 0.03% sodium chloride saline, degreasing solution, and washing water. Process description: the electrolytic generation of nano titanium hydroxide and anatase nanometric titanium dioxide is based on the following process as described in block flow diagram in Fig. 24:

- Titanium sheet is degreased in degreasing solution for removal of oil and greases, then the sheet is washed in running water for complete removal of degreasing solution from the titanium surface.
- Titanium sheet is then neutralized in dilute hydrochloric acid solution, then titanium sheet is hung in electrolytic cell as anode and the direct current is switched on for anodic dissolution for generation of nanometric titanium hydroxide powder.
- The generated nano titanium hydroxide is decanted and filtered then washed for complete removal of sodium chloride and then subjected for drying at 105 °C.
- The complete dried nanometric titanium hydroxide is ground and then subjected for calcination at 600 °C for 4 h to obtain nanometric anatase titanium dioxide which is then cooled to room temperature then packaged in suitable containers.

**Fig. 25:**
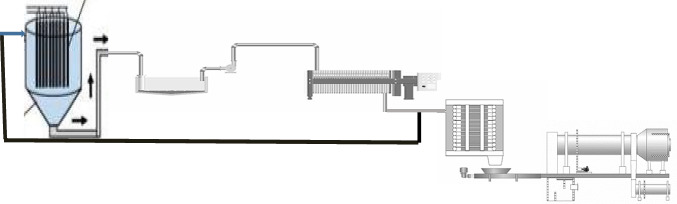
Proposed process instrumental flow diagram for the green electrolytic production of nano titanium hydroxide and nanometric anatase titanium dioxide powder via anodic dissolution of titanium metal flowed by calcination at 600 °C for 4 h

Figure 25 represents the proposed equipment flow diagram of the nano titanium hydroxide and anatase nanometric titanium dioxide production plant which leads to the calculation of the plant cost which is $1.165million as presented in Table 1. The preliminary cost indicators are based on the following assumptions:

- The electrolysis cell and the other used basins of capacity up to 1 m^3^.
- The drier oven is set to 110 °C, and the furnace (for calcination) is set to 600 °C. TiO_2_ purity is 99%, and the conversion rate for the TiO_2_ formation procedure was 85%. Each procedure had 5% losses in the reactor, drying, and calcining.

**Table 1:** Equipment cost

Equipment	Cost
Titanium sheet preparation	
Reactors (Qty: 4) (Carbon steel)	$11,984
TiO_2_ production electrolytic line	
Reactor	$29,424
Centrifugal filter	$217,507
Conveying system	$10,644
Grinder	$35,687
Dryer	$17,300
Packing	$2603
Calcination furnace (carbon steel)	$144,233
Total equipment cost	$427,974
Total direct capital cost	$951,053*
Total fixed capital	$1,403,598
Working capital (15% of fixed capital)	$210,539.7
Total capital cost	$1,614,137.7

^*^Based on chemical industry capital cost estimation, capital cost excludes land cost

All equipment costs were calculated using Peters and Timmerhaus (1990) and updated using ENR cost index. Losses due to the mechanical grindings, drying, calcination, and product packing were 5%. And finally Plant life 20 years, with Plant availability 90%

The preliminary financial indicators of the proposed system are based on the flow sheet shown in Fig. 23. The plant will be working on a zero liquid discharge concept as the chemicals used are recycled along the operation cycle. The power consumption is estimated to be 4.5 kWh/kg titanium feed, for electrolysis cell and total power consumption of 20kWh/1 kg of nanometric titanium dioxide product. The price of electricity is assumed to be $0.15/kWh, and the water cost is estimated to be $1/m^3^. By comparing the results with different published data and other production methods, it is clear that this method of production capital cost is less than the reported cost using liquid-phase synthesis method (Risti et al. 2019) and sol–gel method (Asep et al. 2022). The operating costs are calculated as in Table 2. The operating cost for the production of TiO_2_ is $30.5/kg with depreciation excluding the land price. The current price of nano titanium dioxide is around $240/kg assuming that the commercial price from the factory is about 65% of the end-user price which means that the suggested price will be of $156/kg based on 300 working days annually within the factory that means that the annual sales are US$4.68 million. And the total annual cost is US$0.915 million. The global profit will be US$3.765 million and the net profit after tax will be US$2.824million. The annual cash will be US$2.3million after dividend distribution, the study shows that the internal rate of return (IRR) and the plant will recover its investment within the third quarter of third year based on starting with 30% of the design capacity within the first year, 50% in second year, and reaching maximum capacity in third year of production.

**Table 2:** Different operating cost percentages

Item	Value
Raw materials	As calculated from the process flow sheet
Maintenance*	5% of capital cost
Miscellaneous*	10% of maintained
Overhead*	10% of capital
General overhead*	20–30% of production cost
Taxes	10%

^*^Chemical projects, 2022

## Product quality

The obtained nano titanium hydroxide and anatase nanometric titanium dioxide according to the proposed green electrochemical production technique was subjected to chemical analysis, and it was found that the both products of 99.5% purity could be used in both electronic and cosmetic production and painting industry. The proposed pilot system will consume electricity for the successive production of nano titanium hydroxide and anatase nanometric titanium dioxide of 2000kWh/day. As it is clear from the power consumption values, the total electrical energy consumed during the production target products is about 20,000 kW/ton. The electricity cost in Egypt is about $0.15 per kW. This shows that the energy cost for production of these high value titanium nano-derivatives is cheap and reasonable. The running cost of the proposed system with respect to 1 kg of anatase nanometric titanium dioxide is $30.5 including depreciation and excluding land price which is lower than that reported in literature using the traditional production technologies such as sol–gel which is about $110. Table 3 shows the comparison between the eco-green proposed technology and traditional sol gel and chemical leaching traditional technologies. The comparison indicates that the proposed novel technology is a green cleaner production one. This technology is more environmentally and industrial safer than the traditional production technologies, where no air emission was obtained with regard to the very toxic and polluted air with high concentration of chlorine gas obtained at the traditional production technologies (sol–gel and chemical leaching). Also, the obtained product is of high analytical grade quality while the traditional product is of low-quality grade.

**Table 3:** Comparing proposed technology and traditional technology

Item	Proposed system	Traditional system
Process production time (h)	4	50
Power consumption (kWh)	2000	170
Production cost ($/ton)	30.5	120
Product quality	High quality	High quality in sol gel method only
Sludge	No sludge obtained	High amount
Process corrosively	No corrosion	Very corrosive
Air toxicity	No toxicity	Very toxic chlorine gas
Safety	Very safe	Industrial unsafe

Also, it is observed the presence of sludge in the traditional production process while no sludge at all in the proposed green electrochemical production technology

## Conclusions

Novel and advanced green cleaner electrochemical production of nanometric titanium hydroxide and nanometric anatase titanium dioxide powder available for photo catalysts, cosmetics, and paint production industry were successfully carried out using anodic dissolution of titanium metal in saline solution followed by calcination process for 240 min at 600 °C. The proposed cleaner production system is based on the combination of anodic dissolution, and thermal calcination. The proposed integrated system presents a successful process for obtaining high-quality nanometric titanium derivatives with low capital, and running costs. The proposed system was designed based on optimum operating conditions which are as follows: pH 4, C.D. 65 mA/cm^2^, 25 °C, 150 rpm, electrode gap distance 3 cm, and NaCl 3 g/l for electrolysis time 240 min followed by calcination for 4 h at 600 °C, to reach complete conversion to anatase titanium dioxide nano powder of main particles size of 77 nm with major percentage of 70%. Preliminary design of nanometric titanium obtained derivative production plant with daily capacity of 100 kg was carried out, and economic indicators were calculated which show that the operating cost for the production of nanometric anatase TiO_2_ is $30.5/kg with depreciation excluding the land price. The current price of nano titanium dioxide is around $240/kg, assuming that the commercial price from the factory is about 65% of the end-user price which means that the suggested price will be of $156/kg based on 300 working days annually within the factory that means that the annual sales are US$4.68 million. And the total annual cost is US$0.915 million. The global profit will be US$3.765 million and the net profit after tax will be US$2.824million. The annual cash will be US$2.3million after dividend distribution. Also the study shows that the required investment for plant establishment was US$2.528 million; the study shows that the IRR is 47.509%, and the plant will recover its investment within the second quarter of third year based on starting with 20% of the design capacity within the first year.

## Recommendation

Based on the obtained lab-scale results, it is recommended to carry out detailed technology package for industrial scale cleaner production plant for producing nano metric anatase titanium dioxide powder via the proposed electrochemical technology.

## References

[CR1] Akarsu M, Asiltürk M, Sayilkan F, Kiraz N, Arpaç E, Sayilkan H (2006). A novel approach to the hydrothermal synthesis of anatase titania nanoparticles and the photocatalytic degradation of rhodamine B. Turk J Chem.

[CR2] Ali I, Suhail M, Zied AA, Alwarthan A (2018). Recent advances in syntheses, properties and applications of TiO_2_ nanostructures. Royal Soc Chem RSC Adv.

[CR3] Asep BDN, Hanif NP, Risti R, Suryatno WS (2022). Cost analysis and economic evaluation for TiO2 synthesis using sol-gel method. Moroccan J Chem.

[CR4] Bagheri S, Shameli K, Hamid SBA 2013 Synthesis and characterization of anatase titanium dioxide nanoparticles using egg white solution via sol-gel method. J Chem 2013(848205):5

[CR5] Brunette DM, Tengvall P, Textor M, Thomson P (eds) (2001) Titanium in Medicine: material science, surface science, engineering, biological responses and medical applications. Springer-Verlag, Berlin Heidelberg, p 1019

[CR6] Buraso W, Lachom V, Siriya P, Laokul P (2018) Synthesis of TiO2 nanoparticles via a simple precipitation method and photocatalytic performance. Mat Res Express. 10.1088/2053-1591/aadbf0

[CR7] Cargnello M, Gordon TR, Murray CB (2014). Solution-Phase Synthesis of Titanium Dioxide Nanoparticles and Nanocrystals. Chem Rev.

[CR8] Chuang LCh, Chang HL, Huang SW (2009). Photocatalytic degradation of 4-chlorophenol using prepared TiO2 catalysts. Hung Kuang J.

[CR9] Collazzo GC, Jahn SL, Carreño NLV, Foletto EL (2011). Temperature and reaction time effects on the structural properties of titanium dioxide nano-powders obtained via the hydrothermal method. Braz J Chem Eng.

[CR10] Congxue T, Zhao Z, Jun H, Ni L (2008). Surfactant/co-polymer template hydrothermal synthesis of thermally stable, mesoporous TiO2 from TiOSO4. Mater Lett.

[CR11] Diamanti MV, Del Curto B, Pedeferri MP (2011). Anodic oxidation of titanium: from technical aspects to biomedical applications. J Appl Biomater Biomech.

[CR12] Frazier OH, Dowling RD, Gray LA, Shah NA, Pool T, Gregoric I (2004). The total artificial heart: where we stand. Cardiology.

[CR13] Ghorbanpour M, Hatami M, Hatami M (2015) Activating antioxidant enzymes, hyoscyamine and scopolamine biosynthesis of Hyoscyamus niger L. plants with nano-sized titanium dioxide and bulk application, Acta agriculture Slovenica · April 2015

[CR14] Gratzel M (2003) J Photochem Photobiol C 4:145–153

[CR15] Gupta S, Tripathi M (2012) A review on the synthesis of TiO2 nanoparticles by solution route. Open Chem 10(2). 10.2478/s11532-011-0155-y

[CR16] Hewitt JP (1999) Formulating water-resistant titanium dioxide sunscreens. Cosmet. Toiletries 114: 59. https://chemicalprojects.wordpress.com/2014/05/11/estimation-of-operating-costs/. Accessed June 2022

[CR17] Iwasaki M, Hara M, Ito S (1998). Facile synthesis of nanocrystalline anatase particles from titanyl sulfate. J Mater Sci Lett.

[CR18] Kato A, Takeshita Y, Katatae Y (1989). Preparation of spherical titania particles from inorganic precursor by homogeneous precipitation. Mat Res Soc Symp Proc.

[CR19] Kim SY, Chang TS, Shin ChH (2007). Enhancing effects of ultrasound treatment on the preparation of TiO2 photocatalysts. Catal Lett.

[CR20] Kozlov DV, Paukshtits EA, Savinov EN (2000). The comparative studies of titanium dioxide in gas-phase ethanol photocatalytic oxidation by the FTIR in situ method. Appl Catal B.

[CR21] Labbe M (2008) Photocatalytic degradation of select drinking water pollutants using nanoTiO2 catalyst. A thesis submitted to the faculty of graduate studies through civil & environmental engineering in partial fulfillment of the requirements for the degree of master of applied science at the University of Windsor, Canada

[CR22] Leyens C, Peters M (eds) (2003) Titanium and titanium alloys. fundamentals and applications. WILEY-VCH Verlag GmbH & Co, KGaA, Weinheim

[CR23] Lu ChH, Wen MCh (2008). Synthesis of nanosized TiO2 powders via a hydrothermal microemulsion process. J Alloys Compd.

[CR24] Mahmoodi NM, Arami M (2009) Degradation and toxicity reduction of textile wastewater using immobilized titania nano photocatalysis. J Photochem Photobiol, B 94:20–24 10.1016/j.jphotobiol.2008.09.004 18948013

[CR25] Matijevic E, Budnick M, Meites L (1977). Preparation and mechanism of formation of titanium dioxide hydrosols of narrow size distribution. J Colloid Interface Sci.

[CR26] Mills A, Le Hunte S (1997). An overview of semiconductor photocatalysis. J Photochem Photobiol A Chem.

[CR27] Montgomery JS, Wells MGH (2001). Titanium armor applications in combat vehicles. JOM.

[CR28] Nolph ChA, Sievers DE, Kaewgun S, Kucera CJ, McKinney DH, Rientjes JP, White JL, Bhave R, Lee BI (2007). Photocatalytic study of polymorphic titania synthesized by ambient condition sol process. Catal Lett.

[CR29] Park HK, Moon YT, Kim DK, Kim CH (1996). Formation of mono-disperse spherical TiO2 powders by thermal hydrolysis of Ti(SO4)2. J Am Ceram Soc.

[CR30] Pedraza JA, López R, Martínez F, Páez EA, Gómez R (2009). Effect of chromium doping on visible light absorption of nanosized titania sol- gel. J Nano Res.

[CR31] Peters MS, Timmerhaus KD (1990) Plant design and economics for chemical engineers, (McGraw-Hill Chemical Engineering Series)

[CR32] Phillips LG, Barbano DM (1997). The influence of fat substitutes based on protein and titanium dioxide on the sensory properties of lowfat milks. J Dairy Sci.

[CR33] Ramanavicius S, Ramanavicius A (2020). Insights in the application of stoichiometric and non-stoichiometric titanium oxides for the design of sensors for the determination of gases and VOCs (TiO2−x and TinO2n−1 vs. TiO2). Sensors.

[CR34] Ramanavicius S, Jagminas A, Ramanavicius A (2022). Gas sensors based on titanium oxides (Review). Coatings.

[CR35] Ramanavicius S, Tereshchenko A, Karpicz R, Ratautaite V, Bubniene U, Maneikis A, …, Ramanavicius A (2019). TiO2-x/TiO2-structure based “self-heated” sensor for the determination of some reducing gases. Sensors 20(1):74.10.3390/s20010074 10.3390/s20010074 PMC6982824 31877794

[CR36] Risti R, Nandiyanto ABD, Maulana AC, Oktiani R, Sukmafitri A, Machmud A, Surachman E (2019). Techo-economic analysis for the production of titanium dioxide nanoparticle produced by liquid-phase synthesis method. J Eng Sci Technol.

[CR37] Serpone N, Lawless D, Khairutdinov R (1995). Size effects on the photophysical properties of colloidal anatase TiO2 particles: size quantization versus direct transitions in this indirect semiconductor. J Phys Chem.

[CR38] Shokrollahi A, Gohari M (2016). Preconcentration and determination of titanium in tap water, geological and sunscreen cream samples by CPE-FAAS method. Beni-Suef Univ J Basic Appl Sci.

[CR39] Sivakumar S, Pillai P, Mukundan P, Warrier KGK (2002). Sol–gel synthesis of nanosized anatase from titanyl sulfate. Matt Lett.

[CR40] Štengl V, Bakardjieva S, Murafa N, Houšková V (2008). Hydrothermal synthesis of titania powders and their photocatalytic properties. Ceramics Silikáty.

[CR41] Štengl V, Houšková V, Murafa N, Bakardjieva S (2010). Synthesis of mesoporous titania by homogeneous hydrolysis of titania oxo-sulfate in the presence of cationic and anionic surfactants. Ceramics Silikáty.

[CR42] Sun J, Guo L-H, Zhang H, Zhao L (2014). UV irradiation induced transformation of TiO2 nanoparticles in water: aggregation and photo-reactivity. Environ Sci Technol.

[CR43] Suryanarayana C, Prabhu B (2006) Synthesis of nanostructured materials by inert-gas condensation methods. Nanostructured Mater Process Prop Appl Second Ed 47–90. 10.1016/B978-081551534-0.50004-X

[CR44] Uon L, Santos GN, Chua A (2020). Synthesis and characterization of titanium dioxide nanomaterials via horizontal vapor phase growth (HVPG) technique. ASEAN Eng J.

[CR45] Varner K (2010) State of the science literature review: nano titanium dioxide environmental matters. Final Report Contract No. EP-C-05–059 Task Order No. 94. EPA/600/R-10/089

[CR46] Viana MM, Soares VF, Mohallem NDS (2010). Synthesis and characterization of TiO2 nanoparticles. Ceram Int.

[CR47] Vorkapic D, Matsoukas T (1998). Effect of Temperature and Alcohols in the Preparation of Titania Nanoparticles from Alkoxides. J Am Ceram Soc.

[CR48] Woodrow Wilson International Center for Scholars (2010) The project on emerging nanotechnologies: consumer products inventory, April 16, 2010. http://www.Nano-tech-project.org/inventories/consumer/

[CR49] Xu JH, Dai WL, Li J, Cao Y, Li H, He H, Fan K (2008). Simple fabrication of thermally stable aperture N-doped TiO2 microtubes as a highly efficient photocatalyst under visible light irradiation. Catal Commun.

[CR50] Yang X, Cao C, Erickson L, Hohn K, Maghirang R, Klabunde K (2009). Photo-catalytic degradation of rhodamine B on C-, S-, N-, and Fe-doped TiO2 under visible-light irritation. Appl Catal B.

[CR51] Yang J, Mei S, Ferreira JMF (2003) J Colloid Interface Sci 260:82–88. 10.1016/s0021-9797(02)00190-x 12742037

[CR52] Yang X, Cao C, Erickson L, Hohn K, Maghirang R, Klabunde K (2009a) Photocatalytic activity of multi-doped TiO2 nanoparticles for degredation of rhodamine B, Applied Catalysis B: Environmental 91(3–4):657–662

[CR53] Yanting L, Xiuguo S, Huiwan L, Shaohui W, Yu W (2009). Preparation of anatase TiO2 nano- particles with high thermal stability and specific surface area by alcohothermal method. Powder Technol.

[CR54] Zhang D, Qi L, Ma J, Cheng H (2002). Formation of crystalline nanosized titania in reverse micelles at room temperature. J Mater Chem.

[CR55] Zheng R, Meng X, Tang F (2009). Synthesis, characterization and photodegradation study of mixed- phase titania hollow sub-microspheres with rough surface. Appl Surf Sci.

[CR56] Zucker LG, Darby MR (2004) Nanoscience and nanotechnology opportunities and challenges in California, CHAPTER 2: Formation and transformation of industries: Council on Science and Technology: Sacramento

